# Snail-1 Silencing by siRNA Inhibits Migration of TE-8 Esophageal Cancer Cells Through Downregulation of Metastasis-Related Genes

**DOI:** 10.15171/apb.2018.051

**Published:** 2018-08-29

**Authors:** Maryam Hemmatzadeh, Hamed Mohammadi, Farhad Babaie, Mehdi Yousefi, Mehrdad Ebrazeh, Behzad Mansoori, Dariush Shanehbandi, Behzad Baradaran

**Affiliations:** ^1^Immunology Research Center, Tabriz University of Medical Sciences, Tabriz, Iran.; ^2^Department of Immunology, School of Medicine, Tabriz University of Medical Sciences, Tabriz, Iran.; ^3^Student Research Committee, Tabriz University of Medical Sciences, Tabriz, Iran.; ^4^Department of Laboratory Medicine, Shahid Motahari Hospital, Urmia University of Medical Sciences, Urmia, Iran.

**Keywords:** Esophageal cancer, Snail-1, siRNA, Apoptosis, Metastasis

## Abstract

***Purpose:*** Snail-1 is a transcription factor, which takes part in EMT, a process related to the emergence of invasion and cancer progression. The purpose of this study was to evaluate the effect of Snail-1 silencing on the human esophageal squamous cell carcinoma cell line, namely TE-8, in vitro.

***Methods:*** In this study, transfection of Snail-1 specific siRNA was conducted into TE-8 cells. The relative mRNA expression levels of Snail-1, Vimentin, CXCR4 and MMP-9 and transcription levels of miR-34a and let-7a were investigated by quantitative Real-time PCR. Western blotting was carried out to evaluate the Snail-1 protein level. Migration assay of TE-8 cells was also performed following the presence or absence of Snail-1 specific siRNA. MTT and TUNEL assays were performed to evaluate cell viability after Snail-1 silencing.

***Results:*** It was found that treatment of cancer cells with the Snail-specific siRNA effectively downregulated the expression of Snail-1 in both mRNA and protein levels, and vimentin, CXCR4, and MMP-9 in mRNA level. However, it elevated the transcript levels of miR-34a and let-7a expressions. Furthermore, transfection of cancer cells with the Snail-specific siRNA significantly induced apoptosis in TE8 cells. Moreover, suppression of Snail-1 led to diminished cell migration.

***Conclusion:*** It seems that Snail-specific siRNA can significantly interrupt esophageal cancer cell migration and reduce metastatic-related factors and induce miR-34a and let-7a in vitro. The bottom line is that therapeutic approaches via targeting Snail-1 can be used for ESCC treatment, suggesting that other possible target molecules for ESCC therapy require to be explored.

## Introduction

Esophageal cancer (EC) is considered as the eighth prevalent and the sixth most lethal tumor in the world.^1^ It has been reported that esophageal cancer is the third most common gastrointestinal cancer and 9% of all cancers in north and northeast regions of Iran.^2^ Histologically, the disease is appeared as esophageal squamous cell carcinomas (ESCC) or esophageal adenocarcinoma (EAC) in approximately 95% of patients. The EAC phenotype is almost 50% more common than ESCC in the United States, while it has been reported to be about 20-times less prevalent in Asians.^1,3^ ESCC remains the main subtype of EC in China; however, EAC is the most prevalent form of EC in Western populations.^1^ In spite of recent advancements in surgical techniques, enhanced imaging, and novel chemotherapeutic agents, our apprehension of the disease remains poor, which in part is related to the early invasive and metastatic characteristics of EC cells.^4^ As a consequence, it is paramount to investigate the mechanisms of invasion and metastasis of ESCC. The process of invasion and metastasis of cancer includes several key steps. Among them, the ability to migrate from the primary tumor appears to be the main step. Until now, the most inclusive theory describing how initially quiescent cancer cells obtain metastatic capability is known as the epithelial-mesenchymal transition (EMT).

Snail-1 belongs to the Snail superfamily of zinc-finger transcription factors, which also consist of Snail-2 (Slug) and Snail-3 (Smuc) proteins. This superfamily exerts a function in the survival and differentiation of various cells, which are the two major processes in the mechanobiology of cancers. In humans, chromosome 20q13.2 harbors *snail family transcriptional repressor 1* (*SNAI1*) gene, which codifies a protein consisting of 264 amino acids that exerts transcriptional repressor activity.^5,6^ Snail-1 exerts a pivotal role in the modulation of EMT, in which the suppression of E-cadherin leads to a migratory, mesenchymal phenotype development by epithelial cells. In Snail-1-induced EMT, there is a subsided expression of E-cadherin and claudins, which is accompanied with the upmodulation of vimentin and fibronectin.^7^ Moreover, Snail-1 upregulates MMP-9 and CXCR4, which are central mediators of cancer cells invasion and migration to discrete organs.^7,8^ It has previously been observed that Snail-1 can control the expression of E-cadherin, vimentin, CXCR4, MMP-9, miR-34a, and let-7, which interferes with the EMT process; hence, it seems that Snail-1 may be a crucial modulator during invasive and metastatic behaviors of tumor cells.^9-13^

Here, we assessed the plausible implications of Snail-1 in invasive potential as well as apoptosis of EC cells *in vitro*. We observed that Snail-1 knockdown via small interference RNA (siRNA) inhibited ESCC cell line (TE-8) development and migration and enhanced apoptosis *in vitro*. Additionally, Snail-1 knockdown via siRNA resulted in downregulation of CXCR4 and MMP-9 in TE-8 cell line.

## Materials and Methods

### Cell lines and culture conditions

The ESCC cell line, TE8 (RBRCRCB2098) was provided from cell bank, the RIKEN BioResource Center through the National Bio-Resource Project of the MEXT (RIKEN; Tsukuba, Ibaraki, Japan). The TE-8 cells were maintained in RPMI 1640 culture medium (Sigma-Aldrich, St. Louis, MO, USA), which was supplemented with 10% fetal bovine serum (FBS; Sigma-Aldrich, St. Louis, MO, USA), 1% antibiotics (100 Unit/mL penicillin, and 100 µg/ml streptomycin; Gibco Inc., Paisley, UK.). The culture conditions were 37°C, 95% humidified atmosphere, and 5% CO_2_. Each day, the culture medium changing was conducted, and the cells were passaged after reaching a confluency of 70–90%.

### Transfection of siRNA 

The Snail-1 specific siRNA and the negative control (both Santa Cruz Biotechnology, Inc), namely scrambled control siRNA, were prepared. The Snail-1-specific siRNA involved three different pooled siRNA duplexes sequences (Table 1). TE-8 Cells, at a concentration of 1×10^6^ cells/well, were cultured in 6-well plates and transfected with different concentrations of transfection reagent (4-8 µl) and siRNA (40-80 ρmol) at 70% confluence, according to the manuals provided by the company. The cells were harvested for RNA and protein isolation after 24, 48, and 72 h of transfecting.

### Quantitative RT-PCR analysis (qRT-PCR)

Total RNA content of TE-8 cells was extracted using RNX-PLUS reagent based on the manuals provided by the manufacturer. Afterwards, reverse-transcription of mRNA into complementary DNA (cDNA) was conducted using a universal cDNA synthesis Kit (TAKARA, Japan). On the other hand, to measure the transcript levels of let-7a and miR-34a, the total RNA was reverse-transcribed to cDNA using a Universal cDNA synthesis Kit (Exiqon, Vedbæk, Denmark). After that, 500 ng of cDNA was amplified by Real-time PCR utilizing SYBR Green-1 universal Master mix in a LightCycler® 96 system (Roche, Germany). The transcript level of β-actin, as the housekeeping gene, was also measured. The relative transcription levels were measured based on the comparative C_T_ approach using 2^-RΔCT^ formula described by Schmittgen and Livak.^14^ All qRT-PCR tests were done in triplicate order. The primers applied in real-time PCR quantification are demonstrated in Table 2.

**Table 1:** Snail-1 siRNA sequences

**Cat. number**	**Strand**	**Sequence (3ʹ–5ʹ)**
sc-38398A	Sense	GGACUUUGAUGAAGACCAUtt
	Antisense	AUGGUCUUCAUCAAAGUCCtt
sc-38398B	Sense	CACGAGGUGUGACUAACUAtt
	Antisense	UAGUUAGUCACACCUCGUGtt
sc-38398C	Sense	GCGAGCUGCAGGACUCUAAtt
	Antisense	UUAGAGUCCUGCAGCUCGCtt

**Table 2:** The primers sequences

**Name**	**Type**	**Sequences(3ʹ–5ʹ)**
Snail1	Forward	GGTTCTTCTGCGCTACTGCTG
	Reverse	GTCGTAGGGCTGCTGGAAGG
β-actin	Forward	TCCCTGGAGAAGAGCTACG
	Reverse	GTAGTTTCGTGGATGCCACA
Vimentin	Forward	CAGGCAAAGCAGGAGTCCA
	Reverse	AAGTTCTCTTCCATTTCACGCA
CXCR4	Forward	TCTTCCTGCCCACCATCTACTC
	Reverse	TGCAGCCTGTACTTGTCCGTC
MMP-9	Forward	ATTTCTGCCAGGACCGCTTCTAC
	Reverse	ATCCGGCAAACTGGCTCCTTC
Let-7a	Target sequence	UGAGGUAGUAGGUUGUAUAGUU
miR-34a	Target sequence	UGGCAGUGUCUUAGCUGGUUGU

### Western blot analysis 

Briefly, protein content of cells was isolated by RIPA buffer (25 mM Tris HCl pH 7.6, 150 mM NaCl, 1% NP-40, 1% sodium deoxycholate, 0.1% SDS). Samples containing 50 μg total protein were separated on 12.5% SDS polyacrylamide gel, and then electroblotted to supported PVDF membranes (Roche Diagnostics GmbH). The membranes were then blocked overnight at room temperature with 3% bovine serum albumin (BSA) in TBS-T (1× Tris-Buffered Saline, 0.1% Tween-20). Primary Rabbit polyclonal antibodies were used for detection of Snail-1 (1:500, sc-28199, Santa Cruz Biotechnology) and β-actin (1:3000, monoclonal antibody, Abcam), which were diluted with 0.05% Tween-20 in PBS. After washing several times, the blots were incubated with horseradish peroxidase (HRP)-conjugated goat anti-rabbit secondary polyclonal antibody (1:3000, Santa Cruz Biotechnology). To assess the signals, ECL kit (Roche Diagnostics GmbH) was applied and quantified by NIH ImageJ 1.63 Software.

### MTT assay 

The cytotoxic effects of the additives were elaborated by methyl-thiazol-tetrazolium (MTT) assay Kit (Sigma). Concisely, TE-8 cells were seeded at a density of 15×10^3^ cells/well in 96-well culture, and then treated with the agents and incubated in the humidified CO_2_ incubator. Subsequently, 100 µl of MTT reagent (0.5 mg/ml in PBS) was transferred to each well and the plates were incubated for 4 h. The water-insoluble formazan crystals were constructed during the incubation period that were solubilized by adding 100 µl of the solubilization buffer [Dimethyl sulfoxide (DMSO) + Sorensen buffer] to each well. After 30 min of incubation in the conditions mentioned early, the optical density (OD) of each well was measured at 570 nm using an ELISA reader (Awareness Technology, Palm City, FL, USA). All experiments were done in triplicate order.

### Apoptosis assay

To evaluate apoptosis rate of the cells, the TUNEL assay (Roche Molecular Biochemicals) was done following the manufacturer’s protocol. Brieﬂy, cells were cultivated in 96-well plates (15×10^3^ cells/well), and subsequently, transfected as described above. Afterwards, cells were fixed with 4% paraformaldehyde solution (Sigma-Aldrich, St. Louis, MO, USA) after 48 h, at room temperature in PBS. Then, cells were treated with 0.3% H_2_O_2_-methanol solution and permeabilized with 0.1% Triton X-100 in 0.1% sodium citrate solution for 2 min on ice.

### Scratch-migration assays 

TE-8 cell migration was surveyed using the migration assay. TE-8 cells (4×10^5^ cells/well) were seeded in 24-well plates for 72 h and a scratch was made by using a sterile 100 µl pipette tip across the cell monolayer to make a gap region, at the time of >90% confluency. Cell debris was cleared by washing with PBS. Then 80 ρmol siRNA was used to transfect the TE-8 cells. The gap area at 0, 24, and 48 h after scratching was photographed under the light microscope. Quantification of migration rate was carried out using the NIH Image J software. This assay was repeated independently for three times.

### Statistical analysis 

Representation of the data were done as mean ± standard deviation (SD) from three independent experiments. Differences between groups were analyzed with analysis of variances (ANOVA) with Dunnett’s post-hoc test. *P* values <0.05 were considered as statistically significant level. The statistical analyses and plotting were implemented with GraphPad Prism 6.01 software (GraphPad Software Inc., San Diego, CA).

## Results

### The Snail-1 specific siRNA down-regulated Snail-1 mRNA in human ESCC cell line, TE-8

**Figure 1:**
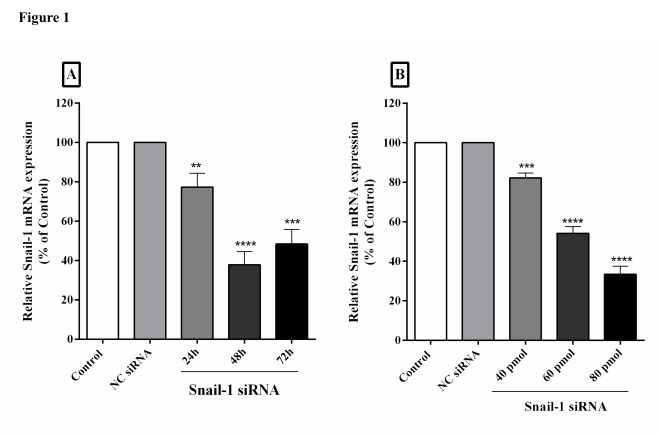
Knockdown of Snail-1 by siRNA in TE-8 cells. (A) Percentage of Snail-1 mRNA expression at three time points of 24, 48 and 72 h. Cells were transfected with 60 ρmol of siRNA. At 24, 48, and 72 h post transfection, mRNA levels were examined by qRT-PCR. Relative mRNA levels were assessed by the qRT-PCR (B) The Snail-1 mRNA expression in doses 40, 60 and 80 ρmol. At 48 h post transfection, mRNA levels were detected by qRT-PCR. The data are represented by mean ± SD (n = 3); **P < 0.001, ***P < 0.001, ****P < 0.0001 versus control. NC; negative control

After exposure to Snail-1 siRNA, TE-8 cells were evaluated by qRT-PCR in 24, 48 and 72 h after transfection. Treating with specific siRNA downregulated Snail-1 mRNA expression significantly (*P*< 0.01; Figure 1A) relative to the control group. After 24, 48, and 72 h since transfection, it was observed that Snail-1 relative mRNA expression was 77.29, 37.85, and 48.37%, respectively. Moreover, the addition of accumulating concentrations of Snail-1 siRNA (40, 60 and 80 ρmol) led to higher downregulation of Snail-1 mRNA (82.15, 54.18 and 33.42%, respectively) (*P*< 0.05, Figure 1B). However, as the negative control, treating with irrelevant siRNA did not affect the suppression of gene expression in TE-8 cells.

### The Snail-1 specific siRNA suppressed Snail-1 protein levels in human ESCC cell line TE-8

**Figure 2:**
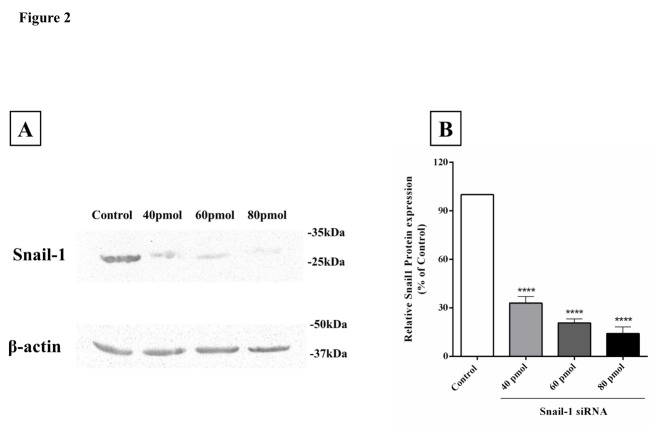
The expression level of Snail-1 protein in TE-8 cells transfected with siRNA. (A) Western blot analysis of Snail-1 in the cells treated with siRNAs. (B) Results indicate the reduction in the transfected cells in a dose of 80 ρmol of siRNA and expression level of each band quantitated by densitometry and intensity of band was normalized to the corresponding β-actin. The data are represented by the mean ± SD (n = 3); ****P < 0.0001 versus control

Western blotting revealed a significant subsiding in Snail-1 protein expression in TE-8 cells transfected with Snail-1-targeting siRNA (Figure 2A and 2B). On the contrary, negative control group demonstrated no significant alteration in protein expression of Snail-1.

### Snail-1 knockdown resulted in cytotoxic effect on TE-8 cells in a dose-dependent manner

**Figure 3:**
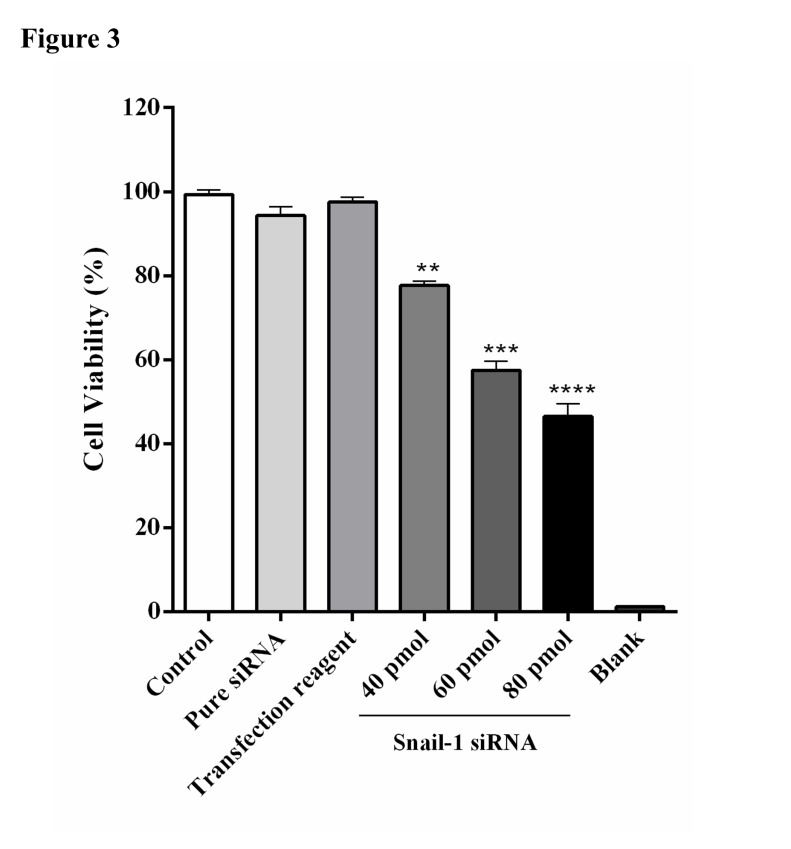
Effect of Snail-1 siRNA on cell survival in TE-8 cell line. Cell survival was assayed by MTT assay 48 h after transfection of three doses of Snail-1 siRNA (40, 60, and 80 ρmol), as described in the methods. At 48 h after transfection with (40, 60, and 80 ρmol), the viability of cells was assessed by MTT assay. The data are represented as mean ± SD (n = 3). **P < 0.001, ***P < 0.001, ****P < 0.0001 versus control

As demonstrated in Figure 3, treating with Snail-1-specific siRNA resulted in increased cytotoxicity. Relative to the control group, Snail-1 siRNA groups demonstrated statistically significant decline in the cell survival rate in a dose-dependent manner.

### Downregulation of Snail-1 enhanced apoptosis in TE-8 cells 

**Figure 4:**
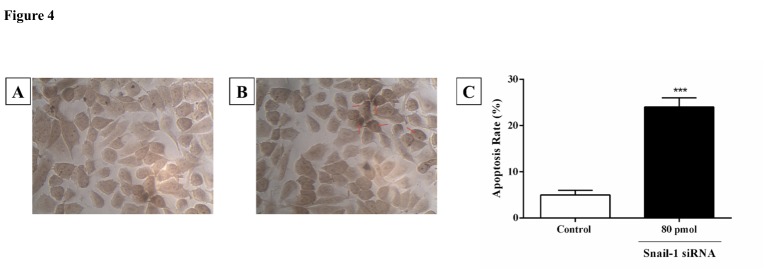
siRNA-mediated targeting of Snail-1 significantly sensitized TE-8 cell lines by stimulation of apoptosis measured by In Situ Cell Death Detection Kit, POD. (A) Untreated siRNA as a negative control. (B) TE-8 cell lines treated with 80 ρmol Snail-1 siRNA (test). (C) Percentage of TUNEL-positive cells in the control and test groups. The results are expressed as mean ± SD (n = 3); ****P < 0.0001 versus control

For studying the pro-apoptotic effects of downregulation of Snail-1, we treated TE-8 cells with Snail-1 specific siRNA. Compared with the control group, transfecting the TE-8 cells with Snail-1 siRNA eventuated in significantly decreased number of surviving cells after 48 h and at the dose of 80 ρmol Snail-1 siRNA (Figure 4A, 4B, and 4C).

### Snail-1 impression on TE-8 cell spread and migration 

**Figure 5:**
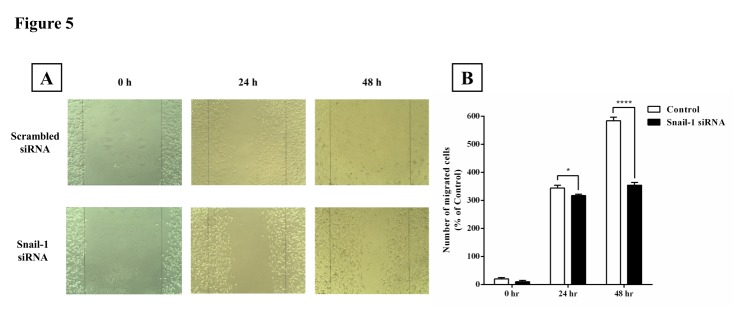
Snail-1 enhances the migration of TE-8 cell line *in vitro*. To determine the Snail-1 effect on cell migration of TE-8 cells, a confluent monolayer of TE-8 cells was scratched and then treated with Snail-1-siRNA. After 0, 24 and 48 h, the scratched area was observed, demonstrating subsided migration ability upon transfecting with Snail-1. (A) Images at 0 h, 24 h, and 48 h after transfection of Snail-1 siRNA and negative control siRNA. (B) The number of migrated cells to the wound sides was‏ evaluated. Data are presented as means ± SD. (n = 3); ****P < 0.0001 versus control

The metastatic potential of TE-8 cells was evaluated using the migration assay *in vitro*. Here, Small RNA interference (siRNA) based strategy was exerted. We measured the ability of transfected cells in filling the gap area over time. The migration assay indicated a significant decline in the total area percentile that was covered upon Snail-1-knockdown relative to the untreated control cells, which filled the gap in 48 h (Figure 5).

### Downregulation of Snail-1 modulated the expression of metastatic-related genes 

**Figure 6:**
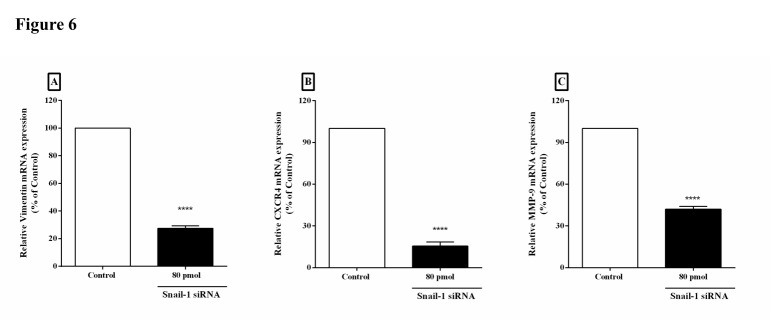
Downregulation of metastatic related genes by RNAi-mediated repression in TE-8 cell line. Vimentin (A), CXCR4 (B), and MMP-9 (C) mRNA levels were reduced after treatment by Snail-1 siRNA in TE-8 cell lines. Levels of vimentin, CXCR4 and MMP-9 expression were downregulated in 80 ρmol Snail-1-treated group compared to the negative control siRNA group. Data are presented as mean ± SD. (n = 3). ****P < 0.0001 versus control

A remarkable reduction in vimentin, MMP-9, and CXCR4 mRNA expressions was observed after *SNAI1* gene knockdown *(P*< 0.0001). The mRNA levels of vimentin, MMP-9, and CXCR4 was considered 100% (NC siRNA) in untreated cells. Relative mRNA expression of vimentin, MMP-9, and CXCR4 genes after knockdown was 27.5%, 42%, and 15.5%, respectively (Figure 6A, 6B, and 6C).

### Upregulation of miR-34a and let-7a following Snail-1-siRNA knockdown 

**Figure 7:**
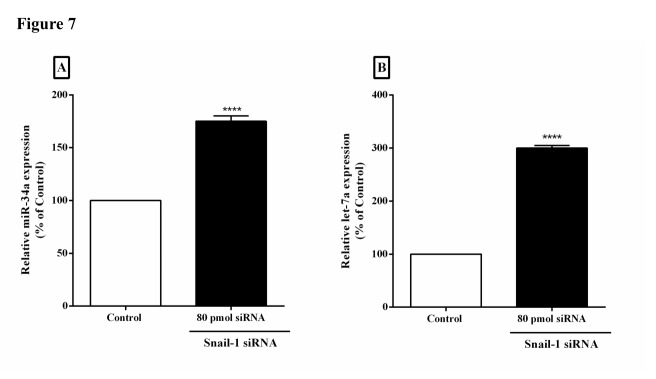
Knockdown of Snail-1 in TE-8 cell lines promotes the miR-34a and let-7a mRNA level. The qRT-PCR analysis of miR-34a (A) let-7a (B) expressions was assessed in TE-8 cells transfected with Snail-1-siRNA. The transcript levels of miR-34a and let-7a significantly were increased following Snail-1 silencing. Results are displayed as the mean ± SD. (n = 3). ****P < 0.0001 versus control

To evaluate the effect of Snail-1 on the expression levels of metastasis-related microRNAs (miRNAs), we carried out qRT-PCR test before and after application of siRNA. Our experiments divulged that miR-34a and let-7a expressions were significantly increased after Snail-1 downregulation (Figure 7A and 7B, *P*<0.0001).

## Discussion

Transforming of epithelial cells into migratory mesenchymal cells is described as EMT, which is the main process mediating chronic inflammation, embryonic growth, and fibrosis, as well as the tumor progression.^15-18^ A bulk of studies has established that Slug/Snail zinc-finger proteins increase the invasive and migratory behaviors of cancer cell through suppressing the adhesive characteristic of epithelial cells.^19-21^ Therefore, Snail can be considered as one of the appealing targets for the progression of pharmaceutical elements. Through blocking Snail, cancer cell metastasis can be interrupted through impairing with the processes, such as EMT and invasive property. The transcription level of Snail, which is a crucial modulator of several controlling mechanisms of EMT, is highly associated with the metastatic trait of tumor cells. Human breast carcinoma has been indicated to require Snail for lymph node metastasis.^22^ Augmented mRNA level of Snail has been detected in metastatic lesions in ovarian cancer.^23^ It was indicated that metastasis process was promoted through Snail-induced EMT after initiation of immunosuppression. Furthermore, cancer development and metastasis were remarkably interrupted through the suppression of Snail.^23,24^ Moreover, repression of molecules with adhesive activity in epithelial cells leads to enhanced migratory characteristics of Snail expressing cancer cells, further conferring the invasion of tumor as well as poor prognosis. Furthermore, metastatic potential of pancreatic tumor cells is thought to be under the impression of Snail expression.^25^ Therefore, Snail can be a pertinent choice to be targeted for preventing the metastasis. Accumulating data indicate that Snail shows a vast spectrum of activities. As a result, Snail can enhance the metastatic trait of esophageal cancer cells and Snail overexpression could prognoses poor clinical outcomes in esophageal cancer patients.

Expression and knockout of the Snail-1 in TE-8 cells were investigated to analyze the functional behavior of these cells. We observed that the suppression of Snail-1 in TE-8 cell line led to downregulation of vimentin, MMP-9, and CXCR4, while miR-34a and let-7a expressions were increased. On the other hand, the intervention caused remarkable reduction of TE-8 cell survival, significant reduction of cell migration, and increased apoptosis rate. Studies established that Snail increased the expression of MMP-9 and vimentin in malignant glioma cell lines,^26^ which was correlated with expression level of CXCR4 in human oral squamous cell carcinoma (OSCC).^8^

The paucity of evidence is now available with respect to the participation of Snail-1 in esophageal cancer etiopathogenesis. Although the expression level of Snail-1 has been investigated in several studies, few studies have been performed about silencing effects on cell migration, apoptosis, and miRNA and gene regulations. Some studies indicated that repression of Snail-1 increased apoptosis in tumor cells,^27^ which are consistent with our findings. It should be noted that‏ we had some limitations in this study. It was better to use Cell Invasion Assays for metastasis. Moreover, it is noteworthy that the downregulation of Snail with a specific siRNA has been associated with an increase in apoptosis, which may also be one of the reasons for a decrease in cell migration.

During EMT, epithelial cells are differentiated to obtain a mesenchymal phenotype, thereby, invasiveness and migratory properties of cells are promoted. In this process, expression of vimentin and fibronectin, as the mesenchymal markers are replaced by the common epithelial markers, such as E-cadherin, Mucin-1, and cytokeratins. Olmeda *et al.* reported that silencing of Snail decreased vimentin in the human breast carcinoma in an *in vitro* study.^22^ Protease enzymes have a critical role in this process, in which extracellular matrix and its components facilitate metastasis of tumor cells to other tissues.^28^ The precise role of proteases, especially MMPs, has been completely verified in metastasis and cancer cell invasion. These enzymes contribute to the process through breaking the connections of connective tissues and digestion of the extracellular matrix components.^29,30^ Miyoshi *et al*. revealed that Snail induced the invasion of hepatoma cells by overexpressing MMP expression, *in vitro*.^31^ Mahabir *et al*. indicated that Snail increased the synthesis of MMP-9 and vimentin in malignant glioma cell lines.^26^ Taki *et al.* demonstrated that there was a correlation between Snail and CXCR4 levels in human OSCC.^8^ In line with these findings, we detected that Snail-1 knockdown culminate in a remarkable reduction in metastasis-related genes, such as vimentin, MMP-9, and CXCR4.

miR-34a is considered as one of the most well-known miRNAs involved in the growth and progression of human tumors.^32,33^ Shi *et al.* reported downmodulation of miR-34a in ESCC cases.^33^ Nie *et al.* demonstrated that upmodulation of miR-34a culminated in increased apoptosis rate and reduced clonogenic formation, whereas it prevented invasion and migration of ESCC cells through repressing MMP-2 and -9 expressions.^34^ We observed that Snail-1 siRNA knockdown resulted in upmodulation of miR-34a and downregulation of MMP-9. These findings imply that siRNA silencing of Snail-1, leading to upmodulation of miR-34a tumor suppressor and downregulation of MMP-1 and MMP-9 metastatic mediators, may interrupt with the migration and invasion of ESCC cells.

Elevated let-7 expression represses the expression of high-mobility group AT-hook 2 (HMGA2), a chromatin remodeling protein that triggers pro-invasive and pro-metastatic genes, such as Snail. Dangi-Garimella *et al.* revealed that pre-miR-let-7a induced the Dicer-processed mature let-7a and subsided HMGA2 and Snail and, therefore, decreased invasion of breast cancer cells.^35^ In our survey, let-7a transcript level was augmented after Snail-1-specific siRNA knockdown. This observation implies that increased level of let-7a might be associated with downregulation of Snail-1 and, thereby, subsided cell migration.

Considering all the facts, Snail-1 mRNA expression was detected in TE-8‏ esophageal cancer cells that was repressed via Snail-1 siRNA, resulting in a remarkable decrease in TE-8 cell survival. Moreover, enhanced apoptosis was identified after Snail-1 knockdown in TE-8 cells. Overall, the outcomes of this study implicate that Snail-1 knockdown utilizing siRNA can significantly interrupt esophageal cancer cell migration and reduce metastatic-related factors, vimentin, CXCR4, MMP-9, and induce miR-34a and let-7a *in vitro*. The bottom line is that therapeutic approaches via targeting Snail can be used for ESCC treatment, suggesting that other possible target molecules for ESCC therapy require to be explored.

## Acknowledgments

This work was supported by a grant of the Immunology Research Center of Tabriz University of Medical Sciences, Tabriz, Iran (grant number: 93/80).

## Ethical Issues

Not applicable.

## Conflict of Interest

The authors declare no conflict of interest.
